# Persistent Hypersomnolence Following Aneurysmal Subarachnoid Hemorrhage: A Case Report

**DOI:** 10.7759/cureus.114425

**Published:** 2026-08-12

**Authors:** Binh Pham, Chris Kocar, Rose Anne M Abe, Talha Memon

**Affiliations:** 1 Sleep Medicine, Southwest Healthcare Medical Education Consortium, Temecula, USA; 2 Family Medicine, Southwest Healthcare Medical Education Consortium, Temecula, USA; 3 Family Medicine, Palmdale Regional Medical Group, Palmdale, USA

**Keywords:** ascending reticular activating system, excessive daytime sleepiness (eds), hydrocephalus, modafinil, mslt, obstructive sleep apnea syndrome, polysomnography (psg), recurrent hypersomnia, secondary central hypersomnia

## Abstract

Persistent excessive daytime sleepiness (EDS) following aneurysmal subarachnoid hemorrhage (SAH) is an underrecognized complication that may impair quality of life and patient safety. We report a 60-year-old man with EDS that had been present for approximately one year and progressively worsened following aneurysmal SAH despite adequately treated obstructive sleep apnea. Overnight polysomnography (PSG) demonstrated well-controlled sleep-disordered breathing, while the multiple sleep latency test (MSLT) revealed severe objective hypersomnolence, supporting a diagnosis of secondary central hypersomnia. Alternative causes of EDS were excluded through clinical evaluation and objective sleep testing. The patient experienced meaningful improvement following treatment with modafinil. This case highlights the importance of considering secondary central hypersomnia in patients with persistent EDS after aneurysmal SAH. It demonstrates the value of PSG followed by MSLT in establishing the diagnosis and guiding management.

## Introduction

Excessive daytime sleepiness (EDS) is a common clinical complaint with a broad differential diagnosis that includes insufficient sleep, sleep-disordered breathing, medication effects, psychiatric disorders, circadian rhythm disorders, and central disorders of hypersomnolence. In patients with persistent EDS despite adequate treatment of obstructive sleep apnea (OSA), further evaluation is warranted to identify alternative or coexisting etiologies. Overnight polysomnography (PSG) is performed to objectively assess sleep architecture and identify sleep disorders that may contribute to EDS. In contrast, the multiple sleep latency test (MSLT) measures physiologic sleep propensity during the day and is the standard objective test for diagnosing central disorders of hypersomnolence [1,2].

Sleep disturbances are common following aneurysmal subarachnoid hemorrhage (SAH) and may persist long after the acute neurologic injury. Previous studies have reported insomnia, fatigue, poor sleep quality, and EDS among survivors, contributing to reduced quality of life and impaired functional recovery [3,4]. However, reports describing objectively confirmed secondary central hypersomnia following aneurysmal SAH remain limited.

The pathophysiology of secondary central hypersomnia following aneurysmal SAH is likely multifactorial. Proposed mechanisms include hypothalamic dysfunction with disruption of orexin-mediated arousal, injury to the ascending reticular activating system (ARAS), cerebral ischemia, and persistent hydrocephalus, all of which may impair the neural networks responsible for maintaining wakefulness [5]. Although the relationship between aneurysm location and secondary hypersomnolence remains incompletely understood, these mechanisms provide a biologically plausible explanation for persistent EDS following aneurysmal SAH.

Given the limited literature describing objectively confirmed secondary central hypersomnia following aneurysmal SAH, additional well-characterized cases are needed to improve recognition of this uncommon complication and to further define the role of objective sleep testing in establishing the diagnosis. This report contributes to the limited literature by illustrating the diagnostic value of overnight PSG and MSLT in confirming secondary central hypersomnia following structural brain injury.

A preliminary version of this work was previously presented as a poster at the 40th Annual Meeting of the Associated Professional Sleep Societies (SLEEP 2026) and published as a conference abstract [6].

## Case presentation

In October 2025, approximately one year after aneurysmal SAH, a 60-year-old man with a medical history of hypertension, hyperlipidemia, depression, hypogonadism, and OSA treated with continuous positive airway pressure (CPAP) presented for evaluation of persistent EDS. His daytime sleepiness had progressively worsened following the aneurysmal SAH, which was complicated by persistent communicating hydrocephalus. Although ventriculoperitoneal shunt placement was recommended by neurosurgery, the patient elected to defer surgical intervention.

The patient reported progressively worsening daytime sleepiness that significantly impaired daily functioning. His Epworth Sleepiness Scale (ESS) score was 15, and he described one episode of falling asleep while stopped at a traffic light [7]. He denied EDS before the aneurysmal rupture and had no history of cataplexy, sleep paralysis, hypnagogic or hypnopompic hallucinations, or other symptoms suggestive of narcolepsy. His habitual sleep duration before evaluation averaged approximately 5.5 hours per night because of work obligations.

On physical examination, the patient was well-appearing and in no acute distress. His body mass index was 31 kg/m², blood pressure was 124/74 mmHg, pulse was 89 beats/minute, and oxygen saturation was 96% on room air. Oropharyngeal examination demonstrated a Mallampati class III airway with macroglossia. Neurological examination demonstrated intact cranial nerves with no motor, sensory, coordination, or gait abnormalities. Because this evaluation occurred during the chronic recovery phase following aneurysmal SAH in the outpatient sleep medicine setting rather than during the acute neurosurgical hospitalization, funduscopic examination and formal assessment for signs of elevated intracranial pressure were not part of the clinical evaluation.

Laboratory evaluation, including a complete blood count, thyroid-stimulating hormone, and vitamin B12 level, was unremarkable. Testosterone remained low, consistent with his known diagnosis of hypogonadism. Pertinent physical examination findings, vital signs, and laboratory results are summarized in Table 1. Before objective sleep testing, modafinil was discontinued, and a sleep diary confirmed an adequate sleep opportunity exceeding seven hours nightly.

**Table 1:** Vital signs and routine laboratory evaluation. Baseline vital signs and laboratory studies obtained during the evaluation of persistent excessive daytime sleepiness. Laboratory testing demonstrated normal complete blood count, thyroid function, and vitamin B12 levels, with low total and free testosterone concentrations.

Parameter	Result	Reference range
Height	5 ft 10 in	-
Weight	216 lb	-
Body mass index	31 kg/m²	18.5-24.9 kg/m²
Blood pressure	124/74 mmHg	-
Heart rate	89 beats/min	60-100 beats/minute
Oxygen saturation	96% (room air)	≥95%
Neck circumference	18 in	-
White blood cell count	7.3 × 10³/µL	3.4-10.8 × 10³/µL
Hemoglobin	15.0 g/dL	13.0-17.7 g/dL
Platelet count	238 × 10³/µL	150-450 × 10³/µL
Thyroid-stimulating hormone	1.16 µIU/mL	0.45-4.50 µIU/mL
Vitamin B12	516 pg/mL	232-1,245 pg/mL
Total testosterone	227 ng/dL	264-916 ng/dL
Free testosterone	5.5 pg/mL	6.6-18.1 pg/mL

Follow-up brain magnetic resonance imaging without contrast demonstrated persistent sequelae of aneurysmal SAH. Imaging showed stable enlargement of the lateral ventricles, persistent prominence of the third ventricle measuring up to 1.2 cm, and moderate dilation of the bilateral temporal horns, consistent with chronic communicating hydrocephalus. Residual periventricular hyperintensity on fluid-attenuated inversion recovery (FLAIR) imaging adjacent to the lateral ventricles had decreased compared with the previous examination, suggesting interval improvement in transependymal edema. Residual superficial siderosis remained along the right cerebral sulci and Sylvian fissure, as well as the medial left posterior cerebral sulci and upper brainstem. Diffusion-weighted imaging demonstrated no evidence of acute infarction, and no acute intracranial hemorrhage was identified.

Overnight PSG followed by MSLT was performed to further evaluate his persistent hypersomnolence. Polysomnographic and MSLT findings are summarized in Tables 2-3, respectively.

**Table 2:** Overnight polysomnography findings. A residual AHI below five events/hour indicates adequate control of OSA. The study demonstrated adequate sleep duration and effective treatment of sleep-disordered breathing. Although the PLMI was elevated, the low PLM arousal index suggested that periodic limb movements were unlikely to account for the severity of the patient’s EDS. AHI, apnea-hypopnea index; CPAP, continuous positive airway pressure; PLM, periodic limb movement; PLMI, periodic limb movement index; REM, rapid eye movement

Parameter	Result
Total sleep time	466 minutes
Sleep efficiency	88.60%
Sleep latency	2 minutes
REM latency	125 minutes
CPAP pressure	10 cm H₂O
Residual AHI	1.4 events/hour
PLMI	19.8 events/hour
PLM arousal index	1.5 events/hour

**Table 3:** MSLT findings. A normal mean sleep latency is greater than eight minutes. The markedly reduced mean sleep latency of two minutes and 30 seconds demonstrated severe objective hypersomnolence. The presence of one SOREMP did not meet MSLT criteria for narcolepsy. MSLT, multiple sleep latency test; REM, rapid eye movement; SOREMP, sleep-onset rapid eye movement period

Nap	Sleep latency (min:sec)	SOREMP	REM latency (min:sec)
1	2:00	Yes	12:30
2	0:30	No	N/A
3	4:00	No	N/A
4	2:00	No	N/A
5	4:00	No	N/A
Mean	2:30		N/A

An MSLT performed the following day demonstrated sleep onset during all five nap opportunities, with a mean sleep latency of two minutes and 30 seconds and one sleep-onset rapid eye movement period (SOREMP). The findings are summarized in Table 3.

Although the MSLT did not meet International Classification of Sleep Disorders criteria for narcolepsy, it demonstrated severe objective hypersomnolence. Given the temporal onset after aneurysmal SAH, adequately treated OSA, adequate sleep opportunity before testing, and absence of another sufficient explanation, the overall findings supported a diagnosis of secondary central hypersomnia. Modafinil was resumed for symptomatic treatment.

Based on the clinical history, neuroimaging findings, objective sleep evaluation, and exclusion of alternative causes, the patient was diagnosed with secondary central hypersomnia following aneurysmal SAH. Modafinil therapy was resumed, resulting in improvement in daytime alertness and functional status.

The differential diagnosis included insufficient sleep syndrome, inadequately treated OSA, medication-induced hypersomnolence, depression, endocrine disorders, narcolepsy, idiopathic hypersomnia, and secondary central hypersomnia. Adequate sleep opportunity was documented before objective sleep testing, OSA was effectively treated with CPAP, and laboratory evaluation did not identify a new metabolic explanation sufficient to account for the severity of his hypersomnolence. Although narcolepsy and idiopathic hypersomnia were considered, the temporal onset of symptoms following aneurysmal SAH together with the persistent structural abnormalities on neuroimaging and objective sleep testing supported a diagnosis of secondary central hypersomnia.

## Discussion

Persistent EDS following aneurysmal SAH presents a diagnostic challenge because neurologic injury, psychiatric comorbidity, medication effects, and coexisting sleep disorders may all contribute to symptoms. Sleep disturbances after aneurysmal SAH commonly include insomnia, sleep-disordered breathing, circadian rhythm disturbances, fatigue, and hypersomnolence [3,4]. A recent scoping review further demonstrated that sleep disturbances following aneurysmal SAH remain underrecognized and are evaluated using heterogeneous methodologies, with relatively few studies employing standardized objective sleep assessments [8]. Accordingly, secondary central hypersomnia confirmed by objective sleep testing remains infrequently reported. This case highlights the importance of considering central disorders of hypersomnolence in patients with persistent EDS despite appropriate treatment of coexisting sleep disorders.

Follow-up brain magnetic resonance imaging demonstrated persistent posthemorrhagic structural abnormalities, including communicating hydrocephalus with ventriculomegaly, temporal horn dilation, residual periventricular FLAIR hyperintensity, and superficial siderosis. These chronic imaging findings support ongoing structural brain injury and provide an anatomic substrate for disruption of central arousal pathways responsible for maintaining wakefulness. Although conventional magnetic resonance imaging cannot directly evaluate the ARAS, the persistent structural abnormalities strengthened the clinical suspicion for secondary central hypersomnia in the appropriate clinical context.

Overnight PSG excluded inadequately treated OSA as the primary cause of the patient's persistent EDS. CPAP therapy effectively controlled sleep-disordered breathing, with a residual apnea-hypopnea index of 1.4 events/hour, while the low periodic limb movement arousal index suggested that periodic limb movements were unlikely to account for the severity of symptoms. Subsequent MSLT demonstrated severe objective hypersomnolence, with a mean sleep latency of 2.5 minutes and sleep occurring during all five nap opportunities. Although one sleep-onset rapid eye movement period was observed, the patient did not meet diagnostic criteria for narcolepsy. Instead, the temporal relationship between symptom onset and aneurysmal SAH, together with persistent structural neuroimaging abnormalities and objective sleep testing, supported a diagnosis of secondary central hypersomnia. These findings underscore the importance of interpreting MSLT results within the broader clinical context rather than relying solely on numerical diagnostic thresholds [1,2].

Previous studies have largely characterized sleep disturbances after aneurysmal SAH using subjective measures of sleep quality, fatigue, and EDS rather than objective evaluation for central hypersomnolence [3,4]. A recent scoping review similarly concluded that sleep disorders following SAH remain underrecognized and are evaluated using heterogeneous methodologies, highlighting the need for standardized objective assessment [8]. In contrast, our patient underwent comprehensive evaluation with overnight PSG followed by MSLT, allowing objective confirmation of secondary central hypersomnia while systematically excluding inadequately treated OSA, insufficient sleep, and other common causes of EDS. A published case of severe hypersomnia following unilateral pulvinar infarction likewise demonstrated that structural injury involving central arousal pathways can produce persistent hypersomnolence [9]. Unlike that patient, who had a focal thalamic lesion, our patient demonstrated diffuse chronic posthemorrhagic abnormalities with persistent communicating hydrocephalus following aneurysmal SAH. These similarities support structural disruption of wake-promoting networks as a shared mechanism, whereas the differing lesion patterns illustrate the heterogeneous neuroanatomical substrates underlying secondary central hypersomnia after cerebrovascular injury.

ARAS plays a central role in regulating arousal and wakefulness, and injury to this network has been increasingly recognized as a mechanism underlying hypersomnolence following cerebrovascular disease. Using diffusion tensor tractography, Jang and Kim demonstrated ARAS injury after aneurysmal SAH [10]. A subsequent case report demonstrated that recovery of hypersomnolence paralleled recovery of the injured ARAS [11]. Although diffusion tensor imaging was not performed in our patient, these observations provide a biologically plausible explanation for the persistent hypersomnolence observed after aneurysmal SAH. Orexin dysregulation has also been proposed as a contributing mechanism following aneurysmal SAH [5]. Although the precise relationship between aneurysm location and secondary hypersomnolence remains incompletely understood, persistent hemorrhagic injury, communicating hydrocephalus, cerebral ischemia, and disruption of diencephalic and brainstem arousal pathways likely contribute to impaired wakefulness following aneurysmal SAH [5,10,11].

This case underscores the importance of a systematic evaluation of persistent EDS following aneurysmal SAH. Neuroimaging, PSG, and MSLT provided complementary evidence supporting secondary central hypersomnia while excluding more common causes of EDS. Recognition of this entity has important therapeutic implications. Although ventriculoperitoneal shunt placement had been recommended for the patient's chronic communicating hydrocephalus, the patient elected to defer surgical intervention. Consequently, treatment with modafinil was directed toward symptomatic management of hypersomnolence rather than correction of the underlying structural abnormality and was associated with meaningful improvement in daytime alertness and functional status [1,2].

This report has several limitations. As a single case report, the findings cannot establish a causal relationship between aneurysmal SAH and secondary central hypersomnia. Diffusion tensor imaging was not performed to directly evaluate the ARAS, cerebrospinal fluid hypocretin levels were not measured, and the anatomical location of the ruptured aneurysm was unavailable in the outside medical records. Nevertheless, the temporal relationship between aneurysmal SAH, persistent structural neuroimaging abnormalities, objective sleep testing, and clinical response to treatment contributes to the limited literature describing secondary central hypersomnia following aneurysmal SAH.

## Conclusions

Persistent EDS following aneurysmal SAH should not be attributed solely to fatigue or coexisting sleep disorders without further evaluation. This case demonstrates the value of overnight PSG followed by MSLT in identifying secondary central hypersomnia after exclusion of other common causes of EDS. Recognition of this underrecognized complication may facilitate timely diagnosis and appropriate treatment, potentially improving patient safety and quality of life.
